# Quality assurance in postgraduate pathology training the Dutch way: regular assessment, monitoring of training programs but no end of training examination

**DOI:** 10.1007/s00428-015-1895-4

**Published:** 2016-01-27

**Authors:** Paul van der Valk

**Affiliations:** Department of Pathology, VU Medical Centre, Amsterdam, The Netherlands

**Keywords:** Pathology, Postgraduate education, Quality assurance, examination

## Abstract

It might seem self-evident that in the transition from a supervised trainee to an independent professional who is no longer supervised, formal assessment of whether the trainee knows his/her trade well enough to function independently is necessary. This would then constitute an end of training examination. Such examinations are practiced in several countries but a rather heterogeneous situation exists in the EU countries. In the Netherlands, the training program is not concluded by a summative examination and reasons behind this situation are discussed. Quality assurance of postgraduate medical training in the Netherlands has been developed along two tracks: (1) not a single testing moment but continuous evaluation of the performance of the trainee in ‘real time’ situations and (2) monitoring of the quality of the offered training program through regular site-visits. Regular (monthly and/or yearly) evaluations should be part of every self-respecting training program. In the Netherlands, these evaluations are formative only: their intention is to provide the trainee a tool by which he or she can see whether they are on track with their training schedule. In the system in the Netherlands, regular site-visits to training programs constitute a crucial element of quality assurance of postgraduate training. During the site-visit, the position and perceptions of the trainee are key elements. The perception by the trainee of the training program, the institution (or department) offering the training program, and the professionals involved in the training program is explicitly solicited and systematically assessed. With this two-tiered approach high-quality postgraduate training is assured without the need for an end of training examination.

## Introduction

In the last decades increasing demands have been made by society on health care providers to document the quality of virtually every aspect of what they do. In the past, this focused largely on medical knowledge and much less (or not at all) on other competencies, notably the ‘softer’ competencies such as cooperation, communication, management skills, leadership, and the likes. There is no doubt that such competencies are very important and significant co-determinants of whether a medical specialist will be a successful professional. Along with the introduction of the CANMEDS competencies [1] attention was requested also for those requirements, although it is not very easy to test and evaluate them. The notion that they are essential but untestable is one of the arguments against summative (exit) examinations, as these tend to overemphasize the medical knowledge aspect of professional competency.

Considering the crucial importance of pathology for the diagnostic process and the subsequent phase of treatment decisions, it is not surprising that pathologists (as all other medical specialists) are required to show that they participate in continuous medical education. Initiatives exist to assess professional aptitude of pathologists through regular examinations, though this has not been widely implemented in Europe (yet). The Pathology branch of the European Union of Medical Specialists (UEMS) has introduced an examination for senior pathology residents that might have served such a purpose. For a variety of reasons, however, its voluntary nature being one of them, this examination as yet has not developed into a generally recognized tool to assess professional competency at a European level [2]. It is taken annually by a small number of non-EU trained pathologists who hope that having passed will facilitate their professional implantation in an EU country. Against this background, it might seem self-evident that in the transition from a supervised trainee to an autonomously functioning professional, formal assessment of whether the trainee knows his/her trade well enough to provide high-quality care is necessary. This would then constitute an end of training examination. Such examinations are practiced in several countries, most notably (but not only) in the USA (the board examinations), the UK (the FRCPath examinations), and in Switzerland [3–5].

Regular (monthly and/or yearly) evaluations are a part of every self-respecting training program, but in the Netherlands these evaluations are formative only: their intention is to provide the trainee with a tool by which they can see whether they are on course with their training. Such evaluations are not used as an instrument to decide whether or not a trainee can continue the training program. The result of an end of training examination has major consequences for the examinee: failing the examination implies that the trainee cannot (yet) be registered as specialist pathologist. Ultimately, repeated failure might result in years of training wasted when the trainee is not registered at all. This is a costly approach towards quality control, both for the registrar and for the involved pathology staff. A training post will have been inefficiently used and considerable frustration will have been generated. This reasoning is not meant to imply that every young physician who entered into a training program should be carried to the finish, in spite of perceived shortcomings. However, this ultimate consequence of an end of training examination justifies critical reflection on the question whether there is sufficient evidence to sustain the hypothesis that it effectively improves the quality of the end-product: a competent pathologist.

## The end of training examination

Little solid scientific evidence exists that an end of training examination improves the quality of the end-product. The following reflections are pertinent to this issue.

- Important differences exist between the countries of the European Union [6]: only a few countries practice end of training examinations and in several countries summative examinations are not part of the training program at all. There is no evidence that pathologists having been educated in the latter countries are less capable than those from countries that require a passing note at an end of training examination. With a sense of pride, I would state that in spite of the lack of such an examination pathologists trained in the Netherlands in general do rather well. Scientific evidence to support the need for a summative end of training examination does not exist.
- Having passed an end of training examination is not a guarantee that the successful candidate disposes of all necessary core competencies, as tends to be emphasized often by senior members of the profession. This statement has some truth, which might be a reason for concern, but it is too general to allow specific action. Every experienced pathologist knows, and those involved in end of training examinations should be acutely aware of this, that it is an illusion to think that the body of knowledge is complete at the end of postgraduate training. Experienced pathologists often tend to compare knowledge and skills of their young colleagues to their own, which is unrealistic and unfair. Years of experience hone skills and reshape and detail knowledge to fit personal needs.
- A general observation which generates some concern is the existence of differences in appreciation depending on the institution in which the examination took place. This is obviously not an issue for a written (multiple choice) test but for examinations which have a practical orientation, essential in order to effectively assess diagnostic competencies, this is a real issue. It raises questions regarding objectivity, reproducibility, and fairness. Different examiners might assess details differently, cases that have to be chosen ad hoc for a practice-oriented examination will vary in complexity. Candidates should have equal opportunities and this will not be easily attained in a practice-oriented examination.
- One should be aware of the fact that an examination is an artifact, in that a diagnostic situation is created that requires a certain aptitude (examination skills) to be successfully brought to the end. The ultimate truth as to what passing an examination is evidence of is that the successful candidate knows how to pass. This does not necessarily translate into a fully capable and optimally functioning pathologist later on. This is not to be taken as a decisive argument against an examination, as coping with a stressful situation is an aptitude that every medical specialist must have.
- Having to pass a test tends to stimulate learning, which is a point in favor of regular examinations.
- A document stating unambiguously that a person has passed a proficiency examination creates a certain degree of confidence in the profession, in the sense that it is open to assessment, however limited the value of any single evaluation moment may be.

## Do alternatives exist?

The above reflections should not be taken as decisive arguments against a summative end of training examination. However, it does not hurt to reflect on alternatives. In the system in the Netherlands, the choice has been made to follow two tracks: (1) not a single testing moment but continuous evaluation of the performance of the trainee in ‘real time’ situations and (2) monitoring of the quality of the offered training program through regular site-visits.

1. Continuous evaluation of trainee performance. Systematic and continuous evaluation of trainee performance must abide by the following rules:
  It must monitor the trainee at multiple occasions. Continuous monitoring would be ideal, but at a regular interval (e.g., monthly) is more practical and efficient. With repeated moments of assessment, there will be less apprehension about a suboptimal performance at a single occasion. Repeated assessments require the involvement of the entire staff involved in teaching, which minimizes the impact of differences in perception between different assessors. The rules followed correspond closely to those practiced in the UK [7] and include equivalents of, e.g., multisource feedback and mini-clinical evaluation exercise. These monthly evaluations can focus on a procedure (autopsy, grossing), on making diagnoses on a set of histology or cytology slides or on a case presentation by the trainee in a multidisciplinary meeting, among others. These frequent ‘short practice evaluations’ as they are called are done by every consultant involved in the training program and, as they are monthly, ideally suited to monitor progress. In a training program of a duration of 5 years, this adds up to a number of evaluation moments between 50 and 60, which allows to cover all areas and provides the temporal frame to perceive how well the trainee progresses. The approach allows asking professionals from different disciplines, even outside the department, to evaluate a trainee. Trainees must store the results of all evaluations in a personal portfolio which is personal property of the trainee, but with trainee consent can be used as evidence of professional aptitude.
  Assessments must appear natural, free of stress beyond the habitual tension induced by the professional activity itself (how a trainee performs under stress might be assessed as such also).
  The assessments will cover both practical aptitude and knowledge. In service learning by doing is of vital importance, but in view of a potential lack of sufficient exposure to rare conditions structured teaching is inevitable in any training program. In the Netherlands, assessment of knowledge and diagnostic capacity is performed using an annual progress test, which has to be taken by all trainees, but has a formative character as it does not result in a pass/fail connotation but provides trainees individualized feed-back on strengths and weaknesses.
  Of all types of assessment the intention should be to provide learning moments to the trainee. The result of an assessment should provide a trainee insight in which areas are comfortably mastered but also where limitations are.
  Assessments will include all domains professional activity and responsibilities (according to CANMEDS criteria)
  Assessment results need to be systematically filed and available for inspection by relevant authorities. Trainees keep an electronic portfolio and so log progress in a variety of domains. The portfolio is private property of the trainee but as a rule trainees will allow pertinent scrutiny of its content. Notably, for final registration by the Committee for the Registration of Medical Specialists the applicant will be requested to show the portfolio, allowing the Committee to evaluate details of the followed training program and results of evaluation all along the program.

This may appear a formidable task, but introduction of this approach in most training programs has been very smooth. Documentation has been somewhat of a problem but with the introduction of an electronic portfolio this has become manageable. A remaining problem is the large amount of information collected and how to use this more effectively. In addition, evaluation and documentation have cast additional administrative burdens on already very busy senior staff.

A final word about evaluation of professional performance once registered as pathologist. Regular proficiency testing is in the making, to preplace the currently used CME credit point system for renewal of registration. If performance on a proficiency test would impact on a ‘renewal or not’ decision it would have the character of an end of training test and the comments listed above would apply.

2. Monitoring of the quality of the offered training program through regular site-visits.

In the system in the Netherlands, this is a crucial element of quality assurance of postgraduate training programs. In the site visit, the essential element is the trainee. The perception by the trainee of the training program, the institution (or department) offering the training program, and the professionals involved in the training program is explicitly solicited and systematically assessed. In the assessment, not only the program director but each member of the training group is scrutinized, based upon the principle that postgraduate education is a team effort.

The following are key characteristics of a site-visit (http://www.knmg.nl/Opleiding-en-herregistratie/Project-MMV-Home/Actueel/nieuwsbericht-1/93185/Handleiding-Visitatie-inclusief-Visitatiewerkdocument-2011.htm):

- Site-visits are conducted as a rule every 5 years
- To reduce time investment both for the group under scrutiny and for those performing the site-visit, existing documentation is used as much as possible. Specific requests for documentation will be designed to provide information also useful in a different context.
- Auto-evaluation is an essential element of the site-visit. Auto-evaluation will reflect the level of compliance of the training program with the legal requirements and also the efforts made to systematically improve quality of training.
- Attention is paid to indicators of continuous monitoring of quality. The idea is to foster continuous attention to quality improvement rather than temporary interest in program quality around the site-visit every 5 years.
- The site-visit committee has a trainee member, preferably an alumnus of the training program visited.
- Site-visits are professional: visitors are specifically trained for this task and their performance is evaluated.

During the site-visit, an essential element to be assessed is whether or not the minimum requirements, as defined by the College of Medical Specialists, are met. This is essentially a ‘pass-fail’ test with the legal requirements as point of reference. Indicators of quality are the following:

- Mission and outcomes
- Training process
- Assessment of trainees
- Staffing
- Training settings and educational resources
- Evaluation of training process
- Governance and administration
- Continuous innovation

In assessing these domains, the site-visit committee, which is composed of five trained physicians including a trainee, uses a combination of quantitative and qualitative parameters. Quantitative parameters, such as the number of biopsies or surgical specimens examined, remain relevant because they reflect acquired experience and sufficient exposure to the corresponding domain. An important additional element is the intention to continuously improve postgraduate training of the training program under scrutiny. This is achieved through implementation of a formalized quality assurance approach according to a ‘Plan-Do-Check-Actualize’ cycle (https://www.deming.org/theman/theories/pdsacycle). As a consequence, in the site-visit report the committee will not only comment on the quality of the ‘end-product’, a medical specialist, but also a systematic approach towards quality improvement.

In view of the variety of aspects of a training program assessed during a site-visit, the visiting committee uses several sources of information allowing different aspects of the program to be assessed. The report of the committee will not only provide an overall appreciation but will specifically address the different domains, e.g., in terms of ‘good’, ‘improvements to be made’, or ‘unacceptable’. The perception of trainees is a crucial source of information.

It is the intention of the procedure to be as much as possible ‘evidence based’. To this end, evaluation is performed using approaches with defined criteria for which scientific evidence of validity exist. The medical specialists responsible for or involved in a training program have active input in the choice of the tools used for quality assessment.

Quality postgraduate education is unthinkable without high quality medical care. As a consequence, quality of care will be also scrutinized during a site-visit. As a rule, approaches and data used for accreditation and certification of (services of) the visited entity will be used.

As a rule, an institution will not offer training for a single specialty but for a large variety of medical specialties. To coordinate, offer educational support for more generic elements of specialty training, foster a quality improving approach, institutions dispose of a central committee for postgraduate education. This body will perform an internal site-visit, usually 1 year before the external site-visit. As training programs are rarely confined within a single institution and often comprise of several institutions within a health care region, regional coordination is an important issue.

## References

[1] van der Lee N, Fokkema JP, Westerman M, Driessen EW, van der Vleuten CP, Scherpbier AJ, Scheele F (2013). The CanMEDS framework: relevant but not quite the whole story. Med Teach.

[2] Van den Tweel JG, Cuvelier C, Freemont AJ (2012). A compulsory examination in pathology: redundant or necessary?. Virchows Arch.

[3] Picarsic J, Raval JS, MacPherson T (2010). United States Medical Licensing Examination step 1 two-digit score: a correlation with the American Board of Pathology first-time taker pass/fail rate at the University of Pittsburgh Medical Center. Arch Pathol Lab Med.

[4] Gray TA. The Royal College of Pathologists Annual Report of Examinations and Assessments undertaken in 2009. http://www.rcpath.org/Resources/RCPath/Migrated%20Resources/Documents/A/annual_report_2009.pdf

[5] Lehr H-A, Moch H, Christen B, Safret A, Gugger M, Rössle M, van Gunten M, Lemoine R, Kurt AM, Caduff R, Arnold W, Singer G, Luscieti P, Bannwart F, Genton CY (2012). Board examination for anatomical pathology in Switzerland: two intense days to verify professional competence. Virchows Arch.

[6] Bosman FT, van den Tweel JG (2009). Unison or cacophony: postgraduate training in pathology in Europe. Virchows Arch.

[7] Bailey D. Ensuring quality in postgraduate medical education: competency testing is the key Virchows Archiv (this issue) 10.1007/s00428-015-1847-z 26374106

